# Matrix Metalloproteinase 13 Is Associated with Age-Related Choroidal Neovascularization

**DOI:** 10.3390/antiox12040884

**Published:** 2023-04-05

**Authors:** Jorge González-Zamora, María Hernandez, Sergio Recalde, Jaione Bezunartea, Ana Montoliu, Valentina Bilbao-Malavé, Sara Llorente-González, Alfredo García-Layana, Patricia Fernández-Robredo

**Affiliations:** 1 Retinal Pathologies and New Therapies Group, Experimental Ophthalmology Laboratory, Department of Ophthalmology, Clinica Universidad de Navarra, 31008 Pamplona, Spain; 2 Retinal Pathologies and New Therapies Group, Experimental Ophthalmology Laboratory, Department of Ophthalmology, Navarra Institute for Health Research, IdiSNA, Clinica Universidad de Navarra, 31008 Pamplona, Spain

**Keywords:** matrix metalloproteinase, age-related macular degeneration, oxidative stress, choroidal neovascularization, angiogenesis

## Abstract

Age-related macular degeneration (AMD) is a leading cause of severe vision loss in older individuals in developed countries. Despite advances in our understanding of AMD, its pathophysiology remains poorly understood. Matrix metalloproteinases (MMPs) have been proposed to play a role in AMD development. In this study, we aimed to characterize MMP-13 in AMD. We used retinal pigment epithelial cells, a murine model of laser-induced choroidal neovascularization, and plasma samples from patients with neovascular AMD to conduct our study. Our results show that MMP13 expression significantly increased under oxidative stress conditions in cultured retinal pigment epithelial cells. In the murine model, MMP13 was overexpressed in both retinal pigment epithelial cells and endothelial cells during choroidal neovascularization. Additionally, the total MMP13 levels in the plasma of patients with neovascular AMD were significantly lower than those in the control group. This suggests a reduced diffusion from the tissues or release from circulating cells in the bloodstream, given that the number and function of monocytes have been reported to be deficient in patients with AMD. Although more studies are needed to elucidate the role of MMP13 in AMD, it could be a promising therapeutic target for treating AMD.

## 1. Introduction

Age-related macular degeneration (AMD), which accounts for 6–9% of all legal blindness cases worldwide, is a major cause of severe vision loss in people aged over 55 years in developed countries [1]. As a multifactorial disorder, AMD is thought to involve intricate interactions between aging, genetic predisposition, and environmental risk factors [2]. New evidence regarding the pathophysiology of macular degeneration, coupled with a rapidly expanding body of knowledge regarding fundamental angiogenesis mechanisms, has resulted in innovative advancements in therapeutic approaches. Angiogenic factors such as vascular endothelial growth factor (VEGF) are crucial for the development of choroidal neovascularization (CNV), and anti-VEGF compounds are an effective AMD treatment [3]. Emerging studies have shown that, in addition to the dysregulation of proangiogenic factors, disturbances in the control of the extracellular matrix (ECM) play a substantial role in the etiopathogenesis of AMD. According to histopathological investigations, AMD causes considerable alterations in ECM elements, such as the collagen and elastic layers in Bruch’s membrane [4]. Matrix metalloproteinases (MMPs) tightly control dynamic ECM metabolism [5], and for this reason, they have been studied in the retina. Limited soluble MMPs (i.e., MMP13) can cleave fibrillar collagens under physiological conditions and could catalyze the initial cleavage in type I collagen, making it susceptible to the gelatinases and other proteases in vascular tissue [6]. Both experimental and clinical evidence suggests the involvement of MMPs in the development of CNV and retinal neovascularization [7,8,9]. Among the MMPs, MMP13 has been linked to the promotion of neovascularization, vascular density, and vascular permeability [10,11,12]. It appears to exert this role through the degradation of extracellular collagen [6]. It has also been shown to indirectly promote neovascularization by promoting VEGF expression [13,14]. Using a murine laser-induced CNV model with wild-type (WT) mice and MMP13-deficient mice (MMP13^−/−^), Lecomte et al. investigated the potential effect of MMP13 in CNV [12]. They discovered that MMP13 deficiency impaired CNV formation, which was completely recovered after WT bone marrow engraftment, showing that WT bone-marrow-derived cells are essential for neovascularization via MMP13 [12]. Moreover, they found an increased MMP13 gene expression in human neovascular membranes (late stages) compared to that in control individuals [12], suggesting a potential role in AMD.

In this study, we characterized the localization of precursor and active MMP13 in cultured retinal pigment epithelial (ARPE-19) cells, in a murine model of laser-induced CNV and measured its total levels in plasma samples from patients with neovascular AMD to confirm its association with the disease.

## 2. Materials and Methods

### 2.1. Cell Culturing

#### 2.1.1. ARPE-19 Culture Conditions and Oxidative Stress Induction in Cultured Cells

ARPE-19 cells (CRL-2302, ATCC, Manassas, VA, USA) (passages 12, 14, and 16) were cultured in 10% fetal bovine serum (FBS, 10270106, Gibco Thermo Fisher, Paisley, UK) in accordance with a previous publication [8]. The culture medium was replaced three times per week. After reaching confluence, the medium was changed to 1% FBS and replaced three times per week for a maximum of 2.5 months. During that time, the cells were not passaged.

ARPE-19 cells were cultured in 24-well plates for 2.5 months as previously mentioned prior to being treated with 600 µM H_2_O_2_ (Panreac, Barcelona, Spain) for 6, 24, 30, and 48 h. Supernatants and lysates were collected at each timepoint and analyzed using Western blot (WB) to determine precursor and active MMP13 protein expressions. Additionally, the effect of H_2_O_2_ on MMP13 expression in ARPE-19 monolayers cultured on coverslips was assessed using immunofluorescence.

#### 2.1.2. MMP13 Localization after Oxidative Stress Induction

To confirm the phenotype, zonula occludens-1 (ZO-1), cytokeratin-18 (CK18), and retinal pigment epithelium-specific 65 kDa protein (RPE65) immunofluorescence were used. The cells were fixed in 4% paraformaldehyde (PFA) (in phosphate buffer, PB) for 5 min at 4 °C. They were then transferred to 1% phosphate buffer saline (PBS) for washing purposes and incubated with a blocking buffer (1% bovine serum albumin, BSA, 0.5% Triton X-100, 0.2% sodium azide, and 1% FBS) for 1 h at 4 °C. The cells were incubated with an Alexa Fluor 594 mouse monoclonal antibody anti-ZO-1 (1:100, 339194, Invitrogen-Life Technologies, Gaithersburg, MD, USA), mouse monoclonal anti-CK18 (1:250, M7010, DAKO, Glostrup, Denmark), mouse monoclonal antibody to precursor and active MMP13 (1:250, MAB3321, Merck, Darmstadt, Germany), and rabbit polyclonal anti-RPE65 (1:250, NB100-355SS, Novus biologicals) at 4 °C overnight. After three 10 min washes, they were incubated with secondary fluorescent antibodies goat anti-mouse (1:250, A11029, Life technologies, Gaithersburg, MD, USA) and donkey anti-rabbit 594 (1:250, R37119, Invitrogen, Carlsbad, CA, USA) for 1 h in the dark. All antibody solutions were based on a blocking buffer. Nuclei were stained with 4′,6-diamidino-2-phenylindole (DAPI). Images were obtained using a laser scanning confocal microscope (LSM800, Zeiss, Oberkochen, Germany).

#### 2.1.3. MMP13 Protein Expression after Oxidative Stress Induction

Precursor and active MMP13 protein expressions were determined in ARPE-19 cell culture supernatants. A total of 60 µL was combined with Laemmli buffer (4× NuPage, Invitrogen, Carlsbad, CA, USA) and boiled for 5 min. The samples were subjected to a 10% sodium dodecyl sulfate polyacrylamide gel electrophoresis (SDS-PAGE) and then transferred onto nitrocellulose membranes (GE Healthcare, Fairfield, CT, USA). Then, a blocking step consisting of 5% skimmed milk (*w*/*v*) and 0.1% Tween-20 (*w*/*v*) in Tris buffer saline (TBS) (1 h, RT) was carried out, and incubation was carried out overnight with a mouse monoclonal anti-MMP13 antibody (1:1000, MAB3321, Merck) at 4 °C. Then, the membranes were subjected to a horseradish peroxidase conjugated goat anti-rabbit antibody (7074S; 1:5000, Cell Signaling Technology Inc., Danvers; MA, USA). SuperSignal^TM^ West Atto Ultimate Sensitivity Chemiluminescent Substrate (A38556, Thermo Fisher Scientific, Rockford, IL, USA) was used to detect the chemiluminescent signals, and Image Quant 400 software (GE Healthcare, Fairfield, CT, USA) was used to measure them. The relative intensities of the immunoreactive bands were determined with ImageQuant TL software (GE Healthcare, Fairfield, CT, USA). To confirm that protein loading was similar across samples, membranes were stained using a Ponceau S red solution (P7170-1L, Sigma, St. Louis, MI, USA) and imaged.

### 2.2. Induction of Choroidal Neovascularization Using Laser in Mice

The study was conducted following the European Community guidelines for ethical animal care and use of laboratory animals (Directive 2010/63/UE) and approved by the University of Navarra Animal Research Review Committee (166-12).

Twelve- to sixteen-week-old wild-type (WT) males (n = 8, C57Bl6/J, Charles River, Wilmington, MA, USA) were used and housed as previously described [8] in standard cages with a 12 h light/dark cycle with food and water ad libitum.

The anesthetics used for the mice were as follows: xylazine (10 mg/kg; Xilagesic 2%; Calier Laboratories, Barcelona, Spain) and ketamine (75 mg/kg; Imalgene 1000; Merial Laboratories, Barcelona, Spain). Tropicamide (3 mg/mL; Alcon Cusí) and phenylephrine (7.8 mg/mL; Alcon Cusí, Barcelona, Spain) eye drops were used to dilate the pupils. Laser-induced CNV lesions (532 nm) were performed similarly to previous published papers [7] by using a digital laser system (Micron IV, Phoenix Research Laboratories, OR, USA). Laser photocoagulation spots (3–4 spots, 250 mW intensity, 0.05 s, and 50 µm size) were made close to the optic nerve. The rupture of Bruch’s membrane (BM) was confirmed by the presence of a bubble immediately after the laser application. Any spot with the presence of a vitreous hemorrhage or bubble absence at the time of laser impact was excluded.

### 2.3. Neovascularization and MMP13 Immunolocalization Using Fluorescence

After euthanasia induction, the eyes of the mice were removed and fixed in a solution of 4% PFA diluted in BP at 4 °C for 1 h. The RPE–choroid–sclera complex was then isolated through microsurgery and bleached by incubating it with H_2_O_2_ (10% in PBS solution) at 55 °C for 1.5–2 h, which allowed for the removal of RPE and choroidal melanocyte pigmentation in whole mounts prior to antibody staining. The whole mounts were immersed in a blocking buffer (PBS, 3% Triton X-100, 0.5% Tween 20, 2% sodium azide, and 1% FBS) for 1 h at 4 °C. Afterwards, biotinylated isolectin (1:240, B-1205; Vector Labs, Burlingame, CA, USA) and mouse monoclonal antibody to precursor and active MMP13 (1:100, MAB3321, Merck, Darmstadt, Germany) were incubated with the flatmounts overnight at room temperature and subjected to PBS washing. Subsequently, the samples were incubated with goat anti-mouse 647 (1:250; 21235; Life Technologies, Carlsbad, CA, USA), streptavidin Alexa Fluor 488 (1:250; S32354; Thermo Fisher, Paisley, UK), and Hoechst 33342 (1:800, H1399, Thermo Fisher, Waltham, MA, USA). Finally, images were captured under a confocal microscope (LSM800; Zeiss, Oberkochen, Germany).

### 2.4. MMP13 Immunolocalization in Human Retina

#### 2.4.1. Processing for MMP13 Localization in Human Tissue

For retinal flat mount immunofluorescence, this study used human donor eyes (n = 3) that did not exhibit macroscopic alterations (e.g., disciform scars, hemorrhages, or pigmentary changes). The eyes were subjected to a 4% PFA (diluted in PB) fixation for 3 h and 2% PFA for 6 days at 4 °C. Following this step, RPE and the retinas were isolated from the optic cup, rinsed with PBS, and exposed to H_2_O_2_ (10% in PBS) at 55 °C for 2 h.

#### 2.4.2. MMP13 Immunolocalization in Human Flatmounted Eyes

The flatmounts were immersed in the blocking buffer for 5 h at 4 °C. They were then exposed for 3 days at RT to mouse monoclonal anti-MMP13 (for precursor and active forms; 1:100, MAB3321, Merck), biotinylated isolectin (1:240, B-1205; Vector Labs, Burlingame, CA, USA), and a goat collagen IV antibody (1:250, 1340-01, Southern Biotech, CA, USA). The samples were washed in PBS and subjected to a 3 h incubation in Alexa Fluor rabbit anti-mouse 647, Alexa Fluor donkey anti-goat 594 (1:250, Life Technologies, Carlsbad, CA, USA), streptavidin Alexa Fluor 488 (1:250; S32354; Thermo ThermoFisher, Paisley, UK), and DAPI (Sigma-Aldrich, St. Louis, MO, USA). Finally, mounting media consisting of PBS-glycerol (1:1) were applied, and a confocal microscope was used for visualization and images capture (LSM800, Zeiss, Oberkochen, Germany).

### 2.5. Patients with Wet AMD

#### 2.5.1. Clinical Samples

In this study, a total of 104 participants were recruited from 2 tertiary referral hospitals, namely, Clínica Universidad de Navarra and Hospital Universitario de Navarra. The sample consisted of 52 patients diagnosed with wet AMD in AREDS category 4 and 52 control participants in AREDS category 1, who were matched for age and sex in a 1:1 ratio. All participants provided written informed consent and were of Caucasian origin. The study was conducted following the ethical guidelines of the Institutional Ethics Review Board of Clínica Universidad de Navarra (2016.092mod1), following the 1964 Helsinki Declaration and its subsequent amendments.

The wet AMD inclusion criterion was a diagnosis of AMD with active subfoveal or juxtafoveolar CNV as defined by optical coherence tomography (OCT) and/or fluorescein angiography (FA) (AREDS category 4). The inclusion criteria for the control group included the absence of RPE abnormalities in the macular area, the absence of drusen or the presence of less than 5 small drusen (≤63 μm), and the absence of any other form of CNV or chorioretinal macular atrophy (AREDS category 1).

The exclusion criteria for both the patient and control groups included an age younger than 55 years, the presence of other diseases characterized by retinal neovascularization, the presence of retinal abnormalities, previous retinal surgery, and over 6 diopters of myopia. A detailed ophthalmologic examination was performed for all cases, which included automatic objective refraction, a visual acuity assessment, slit-lamp biomicroscopy after dilation, color fundus photography, OCT, and/or FA. The control group underwent a visual acuity assessment, a fundus examination after dilation, and a measurement of axial length and refractive error.

#### 2.5.2. Plasma Sample Collection

For the collection of plasma samples, 6 mL venous blood samples were extracted from the peripheral blood of participants using ethylenediaminetetraacetic acid (EDTA)-containing tubes. The collected blood samples were then centrifuged at 1000× *g* for 15 min at room temperature. The extracted plasma was then immediately transferred to a new tube within the first hour of extraction and frozen at a temperature of −80 °C until the time of use.

#### 2.5.3. MMP13 Level Quantification Using ELISA

The total levels of MMP13 present in the plasma samples were measured in duplicate by performing an enzyme-linked immunosorbent assay (ELISA), following the guidelines provided by the manufacturer (ab100605, Abcam, Cambridge, UK). The detection limit for MMP13 was 8.23 pg/mL. Both inter- and intra-coefficients of variation were less than 12%.

### 2.6. Statistics

The normality of the distributions was evaluated using the Kolmogorov–Smirnov test. For quantitative variables that followed a normal distribution, independent Student’s *t*-test was applied for a comparison. The Mann–Whitney U test was used to evaluate variables with non-normal distributions. A one-way ANOVA was used for multiple comparisons followed by Bonferroni post hoc test. The Pearson correlation coefficient was utilized to examine the correlations between variables that had a parametric distribution. Fisher’s exact test was used for a comparison of categorical data between groups. A *p*-value less than 0.05 was considered statistically significant for all tests conducted.

The data are expressed as the mean value with the standard deviation (SD) for the in vitro analysis and as the mean value and the standard error of the mean (SEM) for plasma levels in humans. A statistical analysis was conducted using GraphPad Prism 8.0 software (GraphPad Software, San Diego, CA, USA).

## 3. Results

### 3.1. Phenotypic Characterization of ARPE-19 Cells

**Figure 1:**
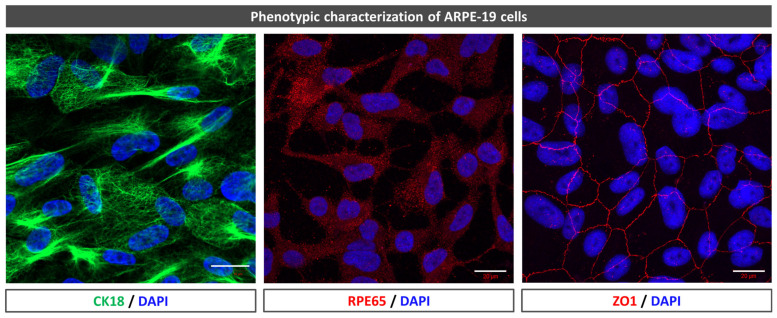
Phenotype confirmation of ARPE-19 cell culture. Immunofluorescence of ARPE-19 cells cultured for 2.5 months labeled with CK18 (**left** image, green), RPE65 (**middle** image, red), and ZO1 (**right** image, red). Nuclei were labeled with DAPI (blue). CK18 = cytokeratin 18; RPE65 = retinal pigment epithelium-specific protein 65 kDa; ZO1 = zonula occludens-1. Scale bar: 20 µm.

We used immunofluorescence to confirm that the phenotype of the ARPE-19 cells used in the experiments corresponded to an RPE phenotype. We confirmed that the ARPE-19 cells expressed CK18, RPE65, and ZO1 (Figure 1).

### 3.2. MMP13 Is Overexpressed under Oxidative Stress Conditions by the RPE

**Figure 2:**
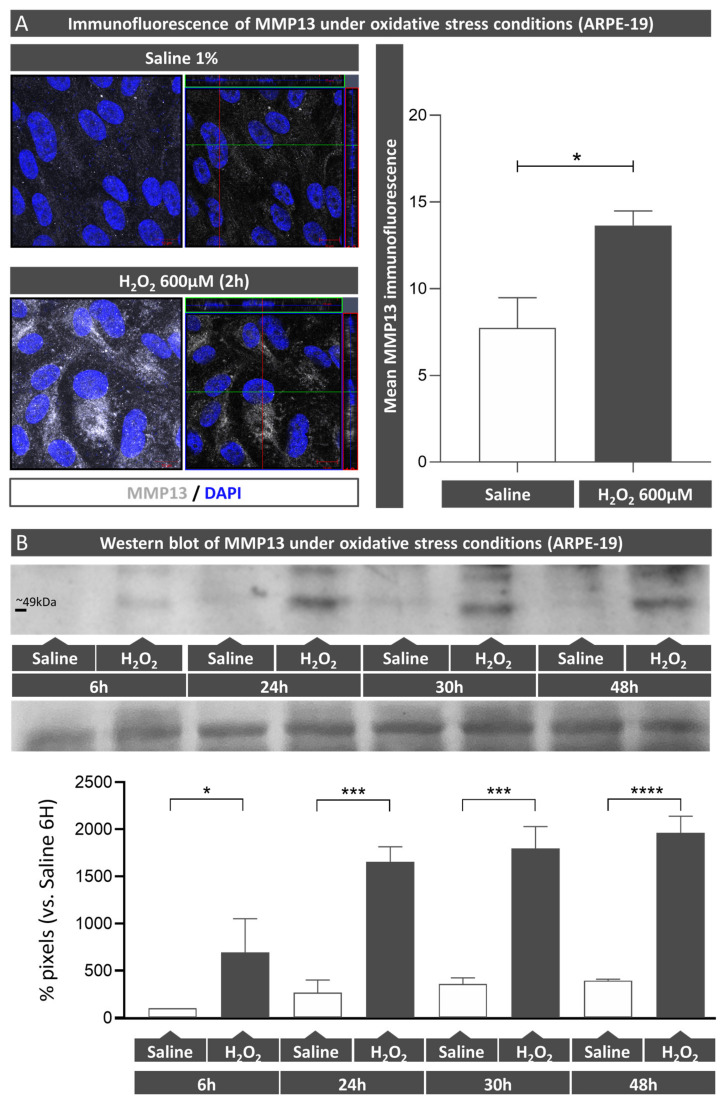
MMP13 localization and overexpression in ARPE-19 cells after oxidative stress induction. (**A**) Immunofluorescence of precursor and active MMP13 under basal conditions with 1% saline and under oxidative stress conditions (600 µM H_2_O_2_ for 2 h). The corresponding orthogonal view of the obtained z-stack is shown next to it. A significant increase in MMP13 expression is observed when subjected to oxidative stress. Mann–Whitney U; * *p* < 0.05 (n = 3). Scale bar: 10 µm. Nuclei are labeled with DAPI (blue). (**B**) WB analysis of MMP13 in ARPE-19 cells exposed to H_2_O_2_ 600 μM at different times (6, 24, 30, and 48 h) vs. saline control 6 h. Ponceau S red is shown below the WB image and confirms the equal loading for each sample. The semi-quantitative histograms of the WB analyses show that MMP13 is expressed under basal conditions over time and that H_2_O_2_ exposition increases its expression. One-way ANOVA followed by Bonferroni. * *p* < 0.05, *** *p* < 0.001, and **** *p* < 0.0001. Bars represent percentage vs. saline 6 h ± S.D. (n = 3). CNV = choroidal neovascularization. MMP13 = matrix metalloproteinase 13. Western blot = WB. MW= molecular weight.

ARPE-19 cells were cultured for 2.5 months and then stained for MMP13. The cells showed a low MMP13 expression under basal conditions (Figure 2A). After inducing oxidative stress in these cells for 2 h with H_2_O_2_, immunofluorescence labeling revealed that MMP13 expression increased significantly more in the induced cells (Figure 2A) than in the non-induced cells (control saline group; *p* < 0.05). The nuclei observed in the oxidative-stress-induced cells were similar to those in the control saline group, suggesting that no apoptosis was present. In addition, cell supernatants from the cells exposed to 600 µM H_2_O_2_ at different time points were subjected to a WB analysis. A significant increase in MMP13 secretion over time under oxidative stress conditions compared to saline at 6 h (*p* < 0.05), 24 h (*p* < 0.001), 30 h (*p* < 0.001), and 48 h (*p* < 0.0001) was observed (Figure 2B).

### 3.3. Murine Retinal Vessels Express MMP13 whereas It Is Absent in the Choroid and the RPE

**Figure 3:**
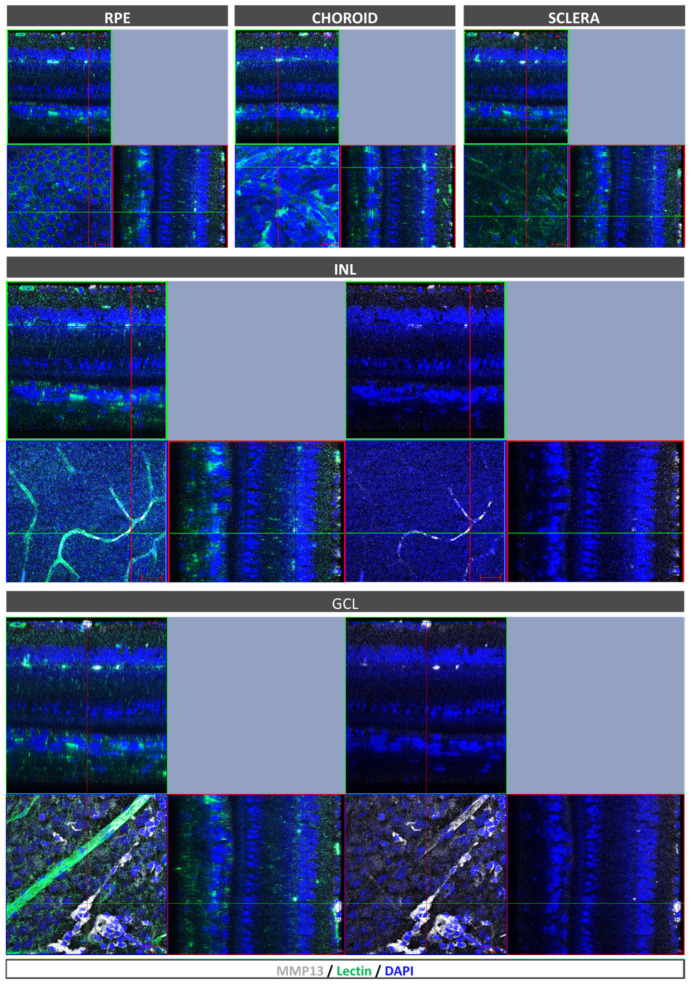
MMP13 characterization in mice posterior pole using immunofluorescence. While no MMP13 expression was observed in the RPE, choroid, or sclera (upper row), the retinal blood vessels on the INL (deep capillary plexus, middle row) and the GCL (superficial capillary plexus, bottom row) showed MMP13 expression, and some lectin-negative cells expressed MMP13. The corresponding orthogonal view of the obtained z-stack is shown on the upper and right-hand side. n = 8. MMP13, white; lectin, green; and DAPI, blue. GCL = ganglion cell layer. INL = inner nuclear layer. MMP13 = matrix metalloproteinase 13. RPE = retinal pigment epithelium. Scale bars: 20 µm.

The immunofluorescence staining of the retinal flat mounts from the WT mice showed that MMP13 was expressed in retinal vessels (lectin-positive), both in the superficial and deep capillary plexuses (Figure 3). The RPE showed no detectable immunofluorescence positivity for MMP13 (Figure 3), and the choroid was lectin-positive but MMP13-negative (Figure 3).

### 3.4. MMP13 Is Present in the RPE and Endothelial Cells from Laser-Induced CNV in Mice

**Figure 4:**
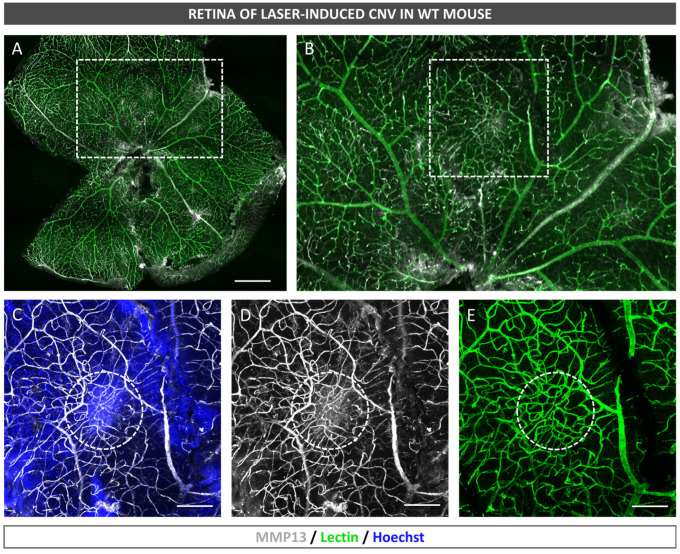
MMP13 expression in laser-induced CNV. WT mouse retina subjected to laser-induced CNV showing MMP13 expression in retinal vessels and diffuse overexpression in retinal parenchyma above the lesions. (**A**) Whole flatmount micrograph; the outlined region represents the magnification shown in (**B**). (**B**) The dotted square represents the magnified area shown in (**C**–**E**), and the retina overlying the CNV area is located in the center (dotted circle). (**C**) MMP13-positive retinal vessels and a cluster of nuclei (blue) above the CNV (dotted circle). (**D**) The cluster of cells shown in (**C**) showed MMP13 expression. (**E**) Retinal vessels positive for lectin (green). n = 8. Hoechst 33342-labeled nuclei, blue; lectin, green; and MMP13, white. EC = endothelial cells. CNV = choroidal neovascularization. MMP13 = matrix metalloproteinase 13. ON = optic nerve. WT = wild type. Scale bars: 500 µm (**A**), 100 µm (**C**–**E**).

**Figure 5:**
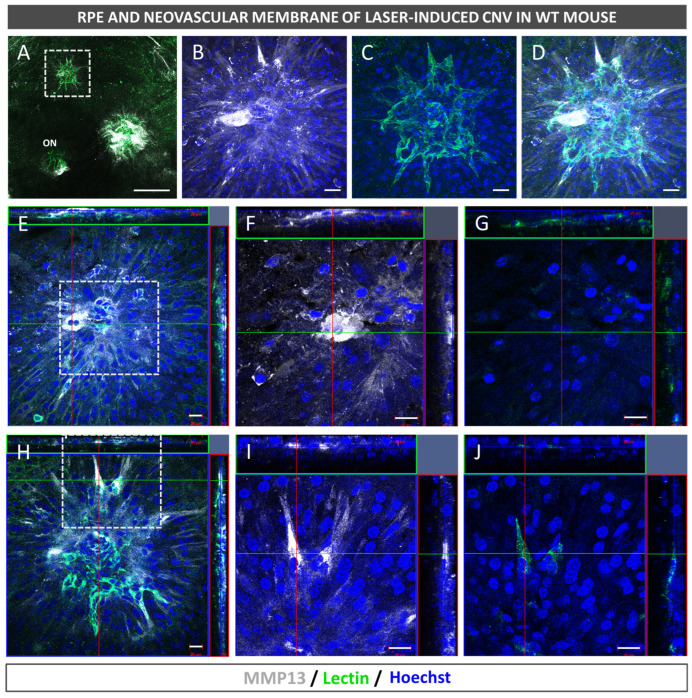
MMP13 is overexpressed in CNV, showing expression in RPE cells and endothelial cells. Representative laser-induced CNV lesions on choroidal flatmounts from WT mice stained with MMP13 (white), lectin (green), and Hoechst 33342 (blue). (**A**) Whole flatmount micrograph with successfully induced CNV. The outlined region represents the CNV shown in (**B**–**J**). (**B**) Magnification of CNV area showing increased MMP13 expression. (**C**) The neovascular membrane is clearly visible and stained with lectin (green). (**D**) Merged image of the MMP13 and lectin staining. (**E**) Orthogonal view of the obtained z-stack at RPE level. (**F**) The RPE showed MMP13 expression, especially in the center of the CNV. (**G**) No lectin-positive cells were observed at this level. (**H**) Orthogonal view of the obtained z-stack at the level of the CNV. (**I**) ECs were MMP13-positive. (**J**) Endothelial cells were lectin-positive (green). n = 8. Hoechst 33342, blue; lectin, green; and MMP13, white. EC = endothelial cells. CNV = choroidal neovascularization. MMP13 = Matrix metalloproteinase 13. ON = optic nerve. RPE = retinal pigment epithelium. WT = wild type. Scale bars: 20 µm (**B**–**J**), 200 µm (**A**).

**Figure 6:**
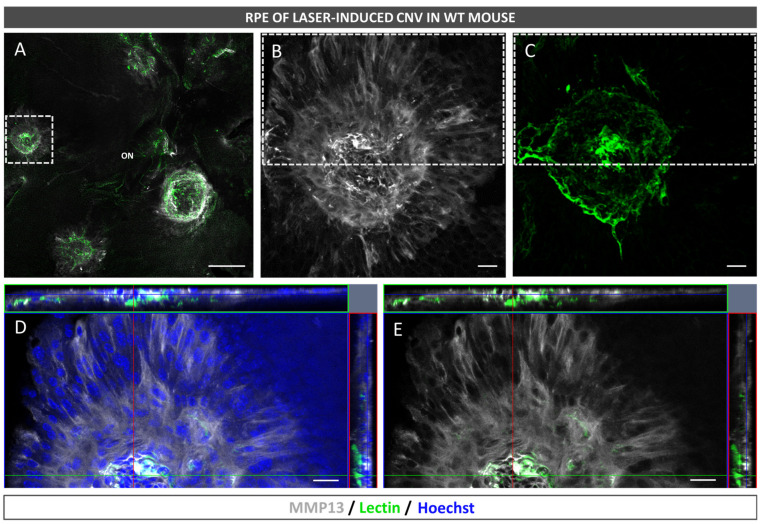
MMP13 is overexpressed in the RPE underlying the CNV. Representative laser-induced CNV lesions on choroidal flatmounts from WT mice stained with MMP13 (white), lectin (green), and Hoechst 33342 (blue). RPE cells underlying the laser-induced CNV mice were positive for MMP13 staining, while the RPE far from this area did not express MMP13. (**A**) Whole flatmount micrograph with three successfully laser-induced CNV lesions; the outlined region represents the magnification in (**B**,**C**). (**B**) MMP13 staining in the CNV area. (**C**) Lectin staining in the CNV area. The outlined region represents the magnification in (**D**,**E**). Panels (**D**,**E**) show that lectin-negative cells, corresponding to RPE cells, expressed MMP13 close to the area of CNV but not outside the lesion. n = 8. Hoechst 33342, blue; lectin, green; and MMP13, white. CNV = choroidal neovascularization. MMP13 = matrix metalloproteinase 13. ON = optic nerve. RPE = retinal pigment epithelium. Scale bars: 200 µm (**A**), 20 µm (**B**–**E**).

Similarly to the non-lasered mice, the retinal vessels in the laser-induced CNV group were MMP13-positive (Figure 4). Diffuse overexpression was observed in the retinal parenchyma above the neovascular lesions of the retinal flat mounts (Figure 4). The endothelial cells of the CNV were lectin- and MMP13-positive (Figure 5). MMP13 expression in the RPE was mainly localized to the CNV area, and an MMP13 signal was not found in the choroid (Figure 6).

### 3.5. Human Retinal Vessels, but Not the Choroid or the RPE, Express MMP13

**Figure 7:**
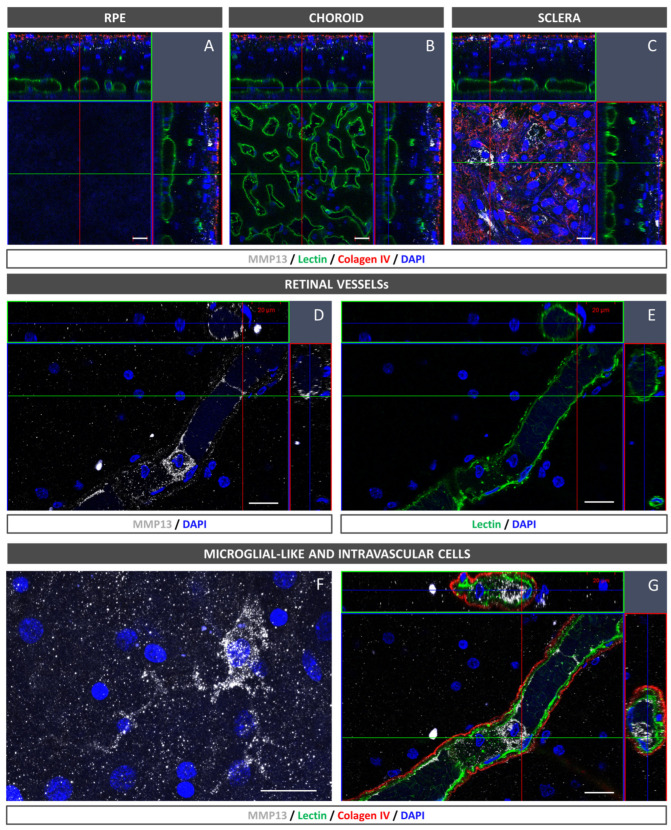
MMP13 immunofluorescence characterization in the human posterior pole. (**A**) The RPE and (**B**) the choriocapillaris did not show MMP13 expression. (**C**) Some cells located in the inner part of the sclera expressed MMP13. (**D**) Retinal vessels showed MMP13 expression, as observed in the z-stack cross-section of the vessel. (**E**) Lectin-positive endothelial cells colocalized with MMP13. (**F**) Some retinal parenchymal cells, predominantly in inner retinal layers, showed a marked MMP13 signal. Some of these cells showed a microglial-like activated morphology. (**G**) MMP13-positive cells were also observed in the lumen of the vessels. n = 3. MMP13, white; lectin, green; collagen IV, red; and DAPI, blue. MMP13 = matrix metalloproteinase 13. ON = optic nerve. RPE = retinal pigment epithelium. Scale bars: 20 µm.

MMP13 expression was characterized in the human eye posterior pole using immunofluorescence. The results show that the RPE and choriocapillaris did not express MMP13 (Figure 7A,B), while it was found to be present in some cells from the inner layers of the sclera (Figure 7C). Moreover, MMP13 expression was detected on the vessel walls (Figure 7D). Lectin-positive endothelial cells colocalized with MMP13 (Figure 7E). Some retinal parenchymal cells, predominantly in the inner layers, showed marked MMP13 expression and an activated microglial-like morphology (Figure 7F). Additionally, MMP13-positive cells were observed in the blood vessel lumen (Figure 7G).

### 3.6. Case–Control Study of MMP13 Plasma Levels

A total of 52 patients with wet AMD were included, with a mean age of 72.70 ± 0.99 years. Of them, 37 were women, and 15 were men. A total of 52 age- and sex-matched controls were selected in a 1:1 ratio, with a mean age of 73.98 ± 0.91. As anticipated, no statistically significant differences in age were observed between the groups (*p* = 0.951). Additionally, non-statistically significant differences regarding the other demographic parameters were found between the groups (Table 1).

**Figure 8:**
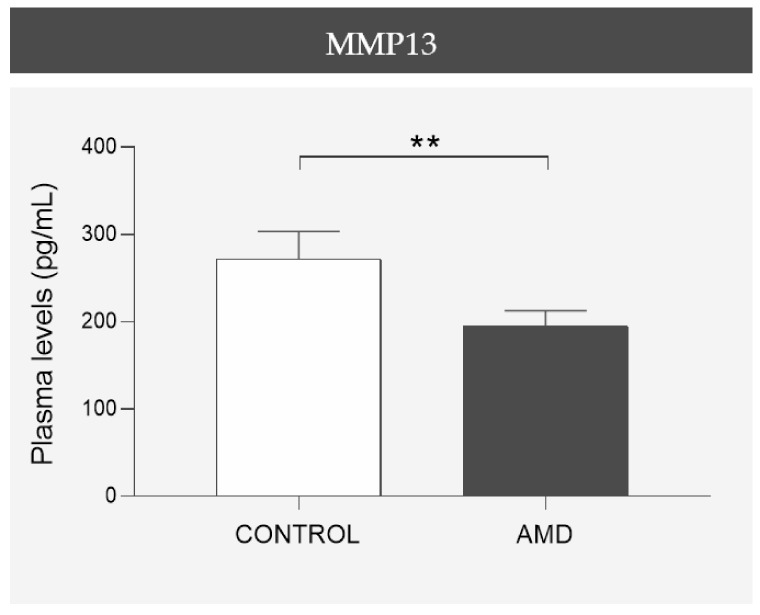
Comparison of mean total MMP13 plasma levels in the age- and sex-matched case–control cohort. A statistically significant difference was found in patients with wet AMD presenting lower plasma levels than controls. AMD = age-related macular degeneration, MMP13 = matrix metalloproteinase 13. ** *p* < 0.01. Error bars correspond to the SEM. Control group, n = 52; AMD group, n = 52. Mann–Whitney U test was used.

The patients with wet AMD showed significantly lower systemic total MMP13 levels when compared with the control group (195.3 ± 17.40 vs. 272.2 ± 31.41 pg/mL, *p* = 0.007) (Figure 8).

An analysis of the correlation between MMP13 and age (Figure S1) revealed no statistically significant difference in either of the groups.

## 4. Discussion

In the present study, we confirmed that cultured ARPE-19 cells, mouse retinas, and human retinas express MMP13. Moreover, we demonstrated that MMP13 is overexpressed under oxidative stress conditions and that it is associated with the area of neovascularization in a murine model of laser-induced CNV. We also observed that normal choroidal vasculature did not express MMP13 in both mice and humans, whereas it was present in our study in CNV. In addition, we found decreased total MMP13 plasma levels in patients with neovascular AMD.

The pathophysiology of AMD, despite being a leading cause of vision loss, is not yet fully understood, and oxidative stress is a main research focus [15]. Some patients with AMD develop choroidal neovascular membranes throughout their disease course. These sprouting vessels grow and cross the BM and the RPE to access the subretinal region. This process requires significant ECM remodeling, which is why MMPs, the main enzymes involved in this process, have generated great interest as possible candidates involved in AMD progression, with MMP2 and MMP9 being the most studied [5]. However, despite the relationship of MMP13 with neovascularization processes [10,11], it has only been described in AMD by one previous study. In this exciting study, Lecomte et al. [12] described that MMP13 deficiency in knock-out mice led to the development of smaller choroidal neovascular membranes. However, they did not evaluate which retinal cellular components were responsible for MMP13 production or whether its expression was altered in AMD. Therefore, determining whether the RPE, the only cellular barrier separating the choriocapillaris from the retina, expresses MMP13 is pertinent. In the present study, we demonstrated that MMP13 expression in ARPE-19 cultures and in mouse and human retinas was barely quantifiable using immunofluorescence and only quantifiable using WB under physiological conditions. Considering that MMP13 is not expressed under basal conditions, we speculate that MMP13 may not have a beneficial role in ameliorating age-related changes in the BM by regulating an adequate turnover of the ECM, which is a suggested role for these groups of proteins [16]. These age-related changes include the deposition of abnormal ECM material, the formation of advanced glycation end products and advanced lipoxidation end products, increased cross-link formation, and increased BM thickness [17]. Therefore, determining whether the basal expression of MMP13 is different in AMD is important to implicate this protein in AMD pathogenesis. In our study, we observed that, in the ARPE-19 cell line subjected in vitro to oxidative stress conditions, which is related to AMD, MMP13 expression noticeably increased. In addition, the RPE underlying the neovascular lesions showed an increase in MMP13 expression, whereas the RPE away from the CNV-induced area maintained a low basal expression. This increase in MMP13 expression in the RPE could partly explain how MMP13 exerts the influence on CNV described by Lecomte et al. (2011). However, this group also demonstrated that an injection of WT BM-derived cells partially restores the deficit in CNV development [12]. This partial restoration suggests that several cell types are responsible for the secretion of MMP13, resulting in CNV promotion. Macrophages are key cells involved in ECM remodeling and, thus, regulate the release and activation of MMPs; in this sense, an increased activation of circulating monocytes has been reported in patients with neovascular AMD [18]. Interestingly, we revealed the presence of cells in the lumen of retinal vessels with a marked expression of MMP13, which could correspond to circulating monocytes or monocytes adhered to the vascular endothelium to migrate to the retinal parenchyma. In addition, we observed cells with an activated microglia-like morphology that also showed elevated MMP13 expressions. These findings are consistent with those of Lecomte et al., who demonstrated that circulating BM-derived cells can modify CNV pathogenesis.

A notable finding in our study was the lack of MMP13 expression in the choriocapillaris of both WT mice and human participants without AMD. This observation contrasts with the expression of MMP13 in retinal blood vessels and suggests differences in MMP13 expression among different endothelial cells within the same organism. The reason for this differential expression is unclear, but it may be related to physiological differences between the choroidal and retinal vasculatures [19]. Choroidal capillaries are fenestrated and have a high permeability, whereas retinal capillaries do not have fenestrations and play a greater role in the blood–retinal barrier. These differences in vascular function and permeability might lead to differences in ECM regulation by MMPs such as MMP13. Interestingly, the endothelium of NVC in mice, despite being derived from choroidal vessels, exhibits high levels of MMP13 expression, indicating that the overexpression is associated with the pathological process. Whether this finding in mice is consistent with that in human samples would be interesting to investigate.

MMP13 may have several mechanisms of action in CNV. As a key interstitial collagenase, it may promote tissue remodeling and EC migration by revealing cryptic binding sites buried in the collagen type IV structure that aid EC adhesion and migration [20]. The angiogenic impact of MMP13 might also require angiogenic factors such as VEGF to be released from these cryptic sites [13]. This would explain the alteration in the pericyte lining that has been well described in mice deficient in MMP13 [12] since an increase in VEGF favors the loss of pericytes [21]. Additionally, MMP13 might participate in a proteolytic cascade to activate MT1-MMP, MMP2, and MMP9 [22].

Interestingly, the partial restoration of impaired CNV in MMP13-deficient mice with BM-derived cells suggests that MMP13 expression may influence disease progression via the circulating peripheral cells of the immune system. Therefore, we wanted to study whether the plasma concentration of circulating MMP13 correlated with the development of neovascular AMD. Surprisingly, compared with the control group, the wet AMD group had significantly lower plasma MMP13 levels. The underlying cause of this decrease in the plasma concentration of MMP13 is difficult to elucidate. It may be a result of decreased diffusion from the parenchyma of the tissues, release from the EC into the bloodstream, or release from circulating cells in the bloodstream (most likely monocytes). In relation to this last possibility, several authors have reported that the number of monocytes is decreased and that monocyte function is deficient in patients with AMD [23,24]. Another possibility is that monocytes may be sequestered in the parenchyma of tissues where they have a role, such as in the eye, explaining the inverse relationship between the tissue and plasma concentrations. This finding is supported by an earlier study that showed that individuals with ischemic central retinal vein occlusion have higher local concentrations of MMPs in their vitreous, despite having lower serum concentrations [25]. Similar findings have been published regarding the lungs, where individuals with idiopathic pulmonary fibrosis have low levels of MMPs in the blood and high levels of MMP expression in the lung parenchyma [26]. The significant decrease in the plasma concentration of MMP13 between the control group and the neovascular AMD group suggests a difference in the profile of cells releasing this metalloproteinase circulating in blood or residing in the parenchyma of other tissues, or the existence of other molecules, including other MMPs, that counteract the overexpression observed in the eye. It would be interesting to elucidate whether this difference is also present in other forms of macular degeneration or whether it is indicative of neovascular pathology. Further information could facilitate early diagnoses, a closer follow-up of patients at risk of neovascular complications, or a differential diagnosis in cases of diagnostic uncertainty.

Our study has the advantage of presenting data from various approaches: cells, a murine model of AMD, and patients with wet AMD.

However, our study has a number of limitations. Some MMP13-positive cells have not been phenotypically characterized; therefore, we cannot state with certainty what these cell types were, since the co-labeling of MMP13 and macrophage or microglia markers was not performed, even though we can infer the most likely cell type on the basis of their location within the retina and their morphology. The correlation between vitreous and/or retinal and plasma concentrations is of great interest; however, the extraction of these samples is an invasive process that is difficult to perform in daily clinical practice and is only ethically admissible within a clinical trial. One constraint of our plasma measurements is that studying total MMP13 expression does not provide information on the activity of the enzyme, as MMP13 is synthesized as a zymogen, which requires proteolytic cleavage to be activated, and our measurement of total MMP13 encompasses both the precursors and the active form.

Future studies are needed to investigate the pathophysiology of MMP13 in CNV and the therapeutic role of its pharmacological or genetic modulation. We believe that, at the cellular level, evaluating the role of MMP13 in angiogenesis models using choroidal ECs would be valuable. Moreover, the expression pattern in patients with neovascular membranes would be interesting to investigate as a complement to our immunofluorescence study in control patients. In addition, as we found a statistically significant relationship with the disease, elucidating the plasma concentration of MMP13 at different stages of the disease, if possible, in paired samples, would also be relevant. In addition, measuring both the precursor and active forms of MMP13 would provide a more complete picture of MMP13 activity and its role in the disease. Finally, the effect of anti-VEGF treatments administered to patients with wet AMD on MMP13 plasma levels can also be investigated in a longitudinal study.

## 5. Conclusions

This study confirmed that MMP13 was expressed in cultured RPE cells and mice retinas. MMP13 expression was also elevated in RPE cells under oxidative stress conditions and in areas of neovascularization in a murine model of laser-induced CNV. Choroidal endothelial cells from both mouse and human retinas did not express MMP13, whereas MMP13 expression was detected in the endothelium of the induced CNV lesions in mice. Plasma MMP13 levels were decreased in patients with neovascular AMD, suggesting a link between the disease and the molecules or cells that regulate its release into plasma. Further investigation is needed to confirm the extent to which MMP13 participates in AMD development.

## Figures and Tables

**Table 1:** Demographic data and clinical characteristics of participants.

	Control (n = 52)	AMD (n = 52)	*p*-Value
Age (years)	72.70 ± 0.99	73.98 ± 0.91	0.342
Female (%)	71%	71%	1.0
HBP (%)	33%	37%	0.83
Smokers (%)	17%	27%	0.345
Dyslipidemia (%)	27%	31%	0.829
Cardiovascular diseases (%)	19%	21%	0.998

AMD = age-related macular degeneration. HBP = high blood pressure. A t-test was used for the comparison of age, and Fisher’s exact test was used for the other variables.

## Data Availability

All data are contained within the article and supplementary.

## Supplementary Materials

The following supporting information can be downloaded at https://www.mdpi.com/article/10.3390/antiox12040884/s1, Figure S1: Correlation of age with the mean plasma levels of MMP13.
