# Supplementary material for: Matrix Metalloproteinase 13 Is Associated with Age-Related Choroidal Neovascularization
Source: Antioxidants (Basel). 2023 Apr 5;12(4):884. doi: 10.3390/antiox12040884 (PMC10135211; doi:10.3390/antiox12040884)
Supplement: Supplementary file 1 [file antioxidants-12-00884-s001.zip › antioxidants-2252056-supplementary.pdf]

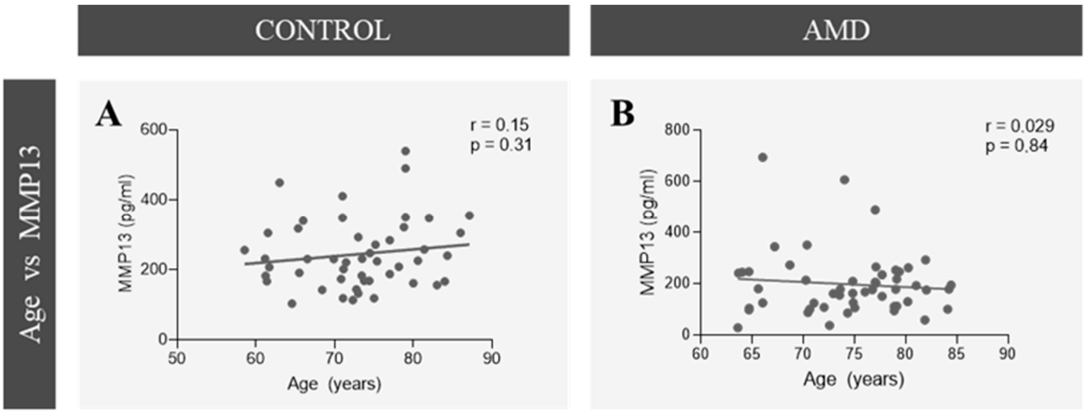

Figure S1. Correlation of age with the mean plasma levels of MMP13. No statistically significant correlation was observed in the studied groups (**A**) Correlation in control group. (**B**) Correlation in AMD group. AMD = Age-related macular degeneration, MMP13 = Matrix metalloproteinase 13. The Pearson's r values are displayed on each graph along with the *p* value of statistical significance.
